# Morphological Description and Potential Geographic Distribution of the Genus *Dolichopus* Latreille (Diptera, Dolichopodidae, Dolichopodinae) in Inner Mongolia, China †

**DOI:** 10.3390/insects14120935

**Published:** 2023-12-08

**Authors:** Xingyang Qian, Xiaolong Ding, Ding Yang, Ning Wang

**Affiliations:** 1 Key Laboratory of Biohazard Monitoring and Green Prevention and Control for Artificial Grassland, Ministry of Agriculture and Rural Affairs, Institute of Grassland Research, Chinese Academy of Agricultural Sciences, Hohhot 010010, China; xyqian1@163.com (X.Q.); dxl15621119356@163.com (X.D.); 2 Department of Entomology, College of Plant Protection, China Agricultural University, Beijing 100193, China

**Keywords:** Diptera, Dolichopodidae, *Dolichopus*, Inner Mongolia, geographic distribution

## Abstract

**Simple Summary:**

The genus *Dolichopus* is a natural enemy of insects. Some of the species are widely distributed, and have the potential to develop into natural insect enemies. Through investigations of the local genus *Dolichopus* in Inner Mongolia, more than 1600 adult long-legged flies were collected, with many of the samples belonging to the subfamily Dolichodinae. Three new *Dolichopus* species were discovered, and another twelve known species were recorded, including the newly discovered subgenus *Hygroceleuthus*, which tremendously enriched the diversity of *Dolichopus* in Inner Mongolia. To have a better understanding of insect diversity and the efficiency of specimen collection in Inner Mongolia, we determined the potential geographic distribution of the genus using a Maximum Entropy (MaxEnt) model.

**Abstract:**

Eight species of *Dolichopus* Latreille were previously recorded in Inner Mongolia. However, there have been only a few studies on their potential distribution. Here, three newly discovered species from Inner Mongolia are described, namely *Dolichopus (Dolichopus) apicimaculatus* sp. nov., *Dolichopus (Dolichopus) jiufengensis* sp. nov., and *Dolichopus (Dolichopus) luae* sp. nov. There were also twelve known *Dolichopus* species that were newly recorded in Inner Mongolia, including the newly recorded subgenus *Hygroceleuthus*. A key to the *Dolichopus* species from Inner Mongolia and the potential geographic distribution of *Dolichopus* in Inner Mongolia were provided. Potential geographic distribution of the genus in Inner Mongolia were determined as well.

## 1. Introduction

Inner Mongolia is part of the Mongolian Plateau, which was created by Qinghai-Tibet Plateau uplift [1,2]. The climate is strongly influenced by plateau uplift, which resulted in Inner Mongolia having a temperate continental climate with relatively high precipitation but low temperatures in the northeast and low precipitation but high temperatures in the southwest [3]. This contrast, in combination with Himalayan orogeny and Quaternary glaciation, significantly influenced the distribution pattern of the plants and animals in Inner Mongolia, which have evolved into groups that are highly adapted to their environment and geographical location.

*Dolichopus* Latreille is one of the largest genera in the subfamily Dolichopodidae with 644 species, of which more than 300 species have been recorded in the Palaearctic region and 72 species are distributed in China [4,5,6]. Previously, eight species have been recorded in Inner Mongolia. However, Inner Mongolia is in the Palaearctic realm, where most of the genus *Dolichopus* is distributed [5]. Therefore, it was predicted that the genus *Dolichopus* likely has more than eight species in Inner Mongolia. In addition, global warming, overgrazing, and human activities have influenced the *Dolichopus* populations and distribution in Inner Mongolia over the years. Thus, global warming is likely to affect the distribution of *Dolichopus* species in the future. Most *Dolichopus* species are predators [5,7,8,9,10,11], and they prefer wet conditions like streams and lakes [5,12].

Currently, research on *Dolichopus* has mainly focused on their morphology and phylogeny, and there have been only a few studies on the potential distribution of *Dolichopus* in Inner Mongolia. Therefore, to further understand the diversity and distribution of *Dolichopus* in Inner Mongolia, a study was conducted on their potential geographic distribution. The regions that were suitable for the genus were also identified. Three new species of *Dolichopus* from Inner Mongolia were described, namely *Dolichopus (Dolichopus) luae* sp. nov., *D. (D.) jiufengensis* sp. nov., and *D. (D.) apicimaculatus* sp. nov. The following twelve species were newly recorded in Inner Mongolia: *D. (D.) aubertini* Parent, 1936, *D. (D.) bigeniculatus* Parent, 1926, *D. (D.) clavipes* Haliday, 1832, *D. (D.) galeatus* Loew, 1871, *D. (D.) geniculatus* Stannius, 1831, *D. (D.) hilaris* Loew, 1862, *D. (D.) longicornis* Stannius, 1831, *D. (D.) ringdahli* Stackelberg, 1929, *D. (D.) tewoensis* Yang, 1998, *D. (D.) zernyi* Parent, 1927, *D. (D.) zhoui* Zhang, Yang & Grootaert, 2004, and *D. (H.) rotundipennis* Loew, 1848. Previously, the low number of recorded species in Inner Mongolia was probably the result of a low number of investigations [5]. The subgenus *Hygroceleuthus* was also newly recorded in Inner Mongolia. A key to *Dolichopus* from Inner Mongolia was also provided. These findings will benefit the further study of *Dolichopus* in Inner Mongolia.

## 2. Materials and Methods

### 2.1. Specimen Collection and Morphology

In this study, the specimens on which this study is based were collected in Inner Mongolia using a sweeping net and stored at the Entomological Museum of China Agricultural University, Beijing, China. The label data of the materials are described below.

The genitalic preparations of the males were made by macerating the apical portion of the abdomen in cold 10% NaOH for 12–15 h. The observations and illustrations were made using a ZEISS Stemi 2000-C (ZEISS, Oberkochen, Germany) stereomicroscope. Photographs were taken with a Canon EOS 77D (Canon, Tokyo, Japan) digital camera through a macro lens. All images were optimized and grouped into plates using Adobe Photoshop CC 2017.

The morphological terminology followed Cumming & Wood [13]. The following abbreviations were used: acr = acrostichal bristle (s), ad = anterodorsal bristle (s), av = anteroventral bristle (s), dc = dorsocentral bristle (s), sc = scutellars, pd = posterodorsal bristle (s), v = ventral bristle (s), LI = foreleg, LII = mid leg, LIII = hind leg, CuAx ratio = length of dm–cu/length of distal portion of CuA.

**Figure 1:**
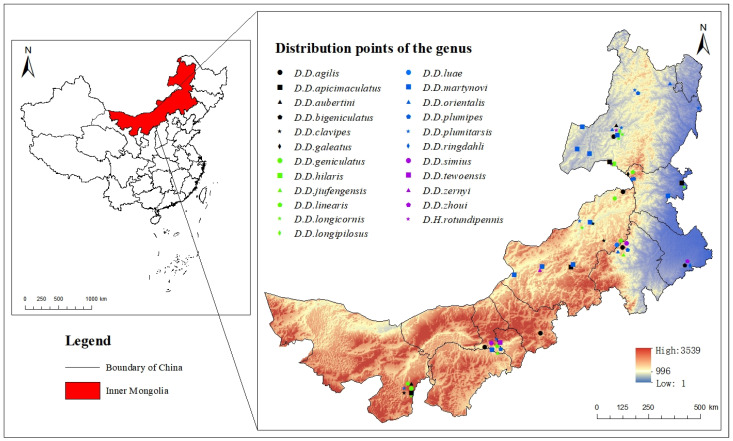
Distribution of *Dolichopus* in Inner Mongolia.

The specimens that were preserved in the Biological Collections of China Agricultural University (1975–2015) were examined and the *Dolichopus* species that were recorded in this study were found to be distributed in different places in Inner Mongolia (Figure 1).

### 2.2. Potential Distribution

The occurrence data of *Dolichopus* were collected during field investigations around Inner Mongolia (Figure 1). Records that did not have an accurate latitude and longitude were excluded. A Maximum Entropy (MaxEnt) model was used to find the probability distribution of the maximum entropy of the known distribution data and related occurrence localities, which can be used to evaluate and model the distribution patterns of species [14]. The MaxEnt model was found to be more accurate than other distribution models when only occurrence data was available [15]. The MaxEnt model was used to predict the potential distribution of *Dolichopus* insects according to the collection records and occurrence records of *Dolichopus* in Inner Mongolia, which provided a potential distribution for *Dolichopus* in Inner Mongolia and more information for species collection. The MaxEnt model was constructed using the occurrence records in Figure 1, and it was based on climate, vegetation type, soil type, and elevation variables.

**Figure 2:**
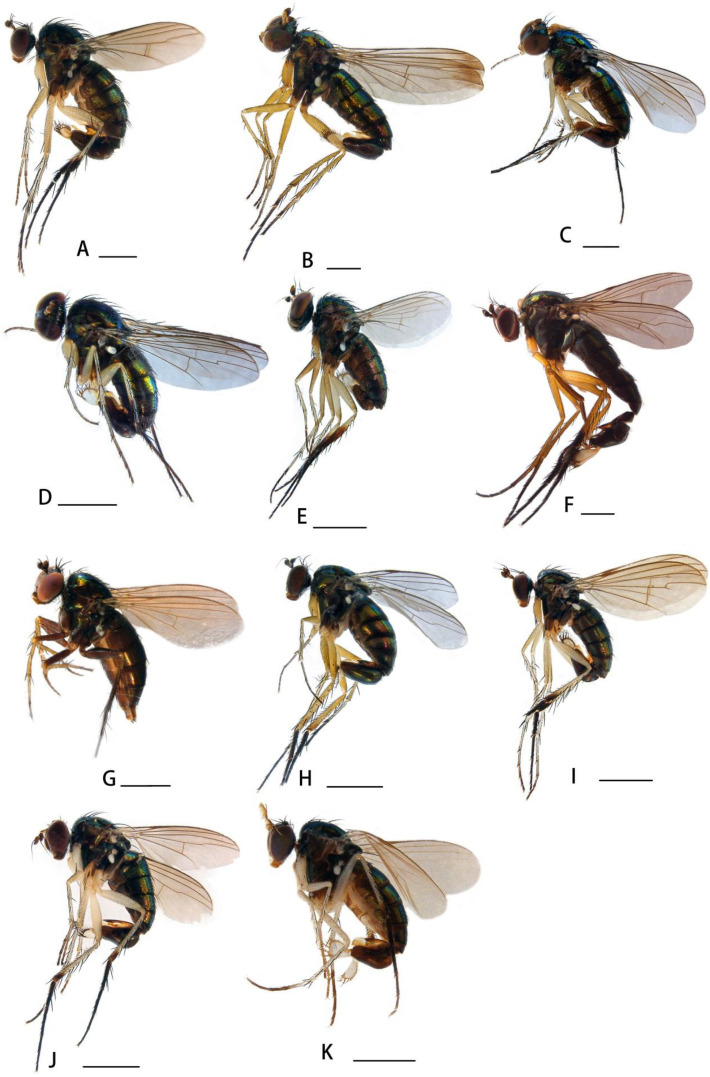
(**A**–**K**) Habitus, lateral view. (**A**) *Dolichopus (D.) agilis* Meigen, 1824, male (**B**) *Dolichopus (D.) apicimaculatus* sp. nov., male (**C**) *Dolichopus (D.) aubertini* Parent, 1936, male (**D**) *Dolichopus (D.) bigeniculatus* Parent, 1926, male (**E**) *Dolichopus (D.) clavipes* Haliday, 1832, male (**F**) *Dolichopus (D.) galeatus* Loew, 1871, male (**G**) *Dolichopus (D.) geniculatus* Stannius, 1831, female (**H**) *Dolichopus (D.) hilaris* Loew, 1862, male (**I**) *Dolichopus (D.) jiufengensis* sp. nov., male (**J**) *Dolichopus (D.) linearis* Meigen, 1824, male (**K**) *Dolichopus (D.) luae* sp. nov., male. Scale bars: 1 mm.

The climatic variables that were used for modeling were obtained from the WorldClim Database (http://www.worldclim.org/, accessed on 20 March 2023). Nineteen climate variables that were recorded between 1950 and 2000 were used (Bio1-19). Digital elevation models were downloaded from Geospatial Data Cloud (https://www.gscloud.cn, accessed on 19 March 2023, Chinese Academy of Sciences). Image resolution was set at 2.5 arc min (about 5 km at the equator). Data on the vegetation and soil types were obtained from a national database, which was updated in 2008 and 2015 and had a resolution of 2.5 arc min (about 5 km at the equator). River data were obtained from the digital elevation model. The data are shown in Figure 2.

Next, MaxEnt (https://biodiversityinformatics.amnh.org/open_source/maxent/, accessed on 7 July 2022) was used to clarify the potential distribution of *Dolichopus*. The model was cross-validated using a 25% subset of randomly selected data points for testing, and 75% of the points were reserved for training. The jackknife method modeled the contribution of the eco-environmental variables to the distribution of the genus *Dolichopus*. Variables with a correlation coefficient (r) > 0.7 were considered to make a significant contribution to the model. A receiver operating characteristic (ROC) curve analysis was used to evaluate the performance of the model in predicting the distribution of the genus *Dolichopus* in China. The model accuracy (area under the curve) was 0.50 with graphical environmental variable averages of 0.80–0.90 (good) and 0.90–1 (excellent).

## 3. Results

### 3.1. Description of New Species

Members of *Dolichopus* can be identified by the following features: hind tarsomere 1 with strong dorsal bristles; wing with costal callus indistinct to long and thick, M weakly bent without rudimentary M_2_ or strongly bent at a right angle with rudimentary M_2_; male genitalia with cercus usually rather large and nearly quadrate with distinct finger-like marginal denticles and bristles [5,16].

#### 3.1.1. *Dolichopus agilis* Meigen, 1824

Figure 2A

*Dolichopus agilis* Meigen, 1824: 97. Type locality: not given [17].

*Dolichopus agilis* Meigen, 1824. Yang et al. 2010: 485 [5].

**Diagnosis.** Postpedicel black. Fore tibia without apico-ventral bristle; hind tibia black at basal 1/3. Costal callus long and strong; M weakly bent without rudimentary M_2_.

**Specimens examined.** 1 male 3 females, CHINA, Inner Mongolia, Saihanwula, 1070m, 2013.VII.22, Xiumei Lu (CAU); 3 males 2 females, CHINA, Inner Mongolia, Hulun Buir, Hailaerdaqiao, 2014.VII.19, Yanan Lv (CAU); 1 male, CHINA, Inner Mongolia, Ulanqab, 1978.VII.8, Heming Chen (CAU).

**Distribution.** China (Inner Mongolia, Hebei, Ningxia, Gansu).

#### 3.1.2. *Dolichopus apicimaculatus* sp. nov.

**Figure 3:**
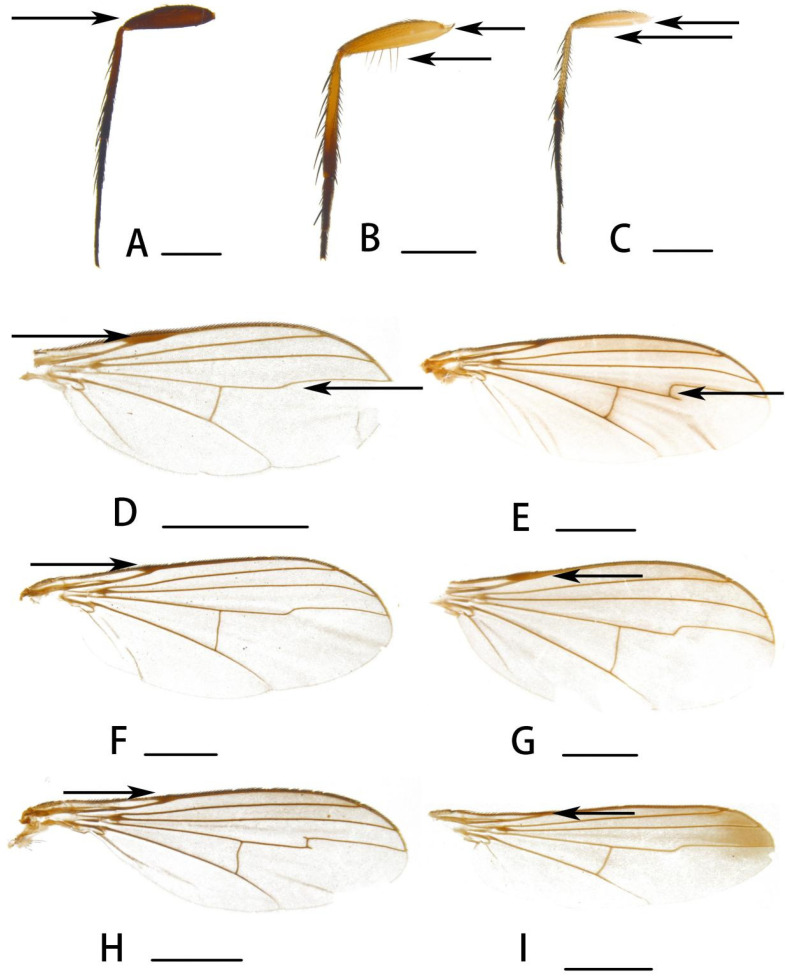
(**A**–**I**) Specific distinguishing features for the key. (**A**) *Dolichopus (D.) galeatus* Loew, 1871, hind leg (**B**) *Dolichopus (D.) martynovi* Stackelberg, 1930, hind leg (**C**) *Dolichopus (D.) linearis* Meigen, 1824, hind leg (**D**) *Dolichopus (D.) longicornis* Stannius, 1831, wing (**E**) *Dolichopus (D.) martynovi* Stackelberg, 1930, wing (**F**) *Dolichopus (D.) orientalis* Parent, 1927, wing (**G**) *Dolichopus (D.) jiufengensis* sp. nov., wing (**H**) *Dolichopus (D.) bigeniculatus* Parent, 1926, wing (**I**) *Dolichopus (D.) apicimaculatus* sp. nov., wing. Scale bars: 1 mm.

**Figure 4:**
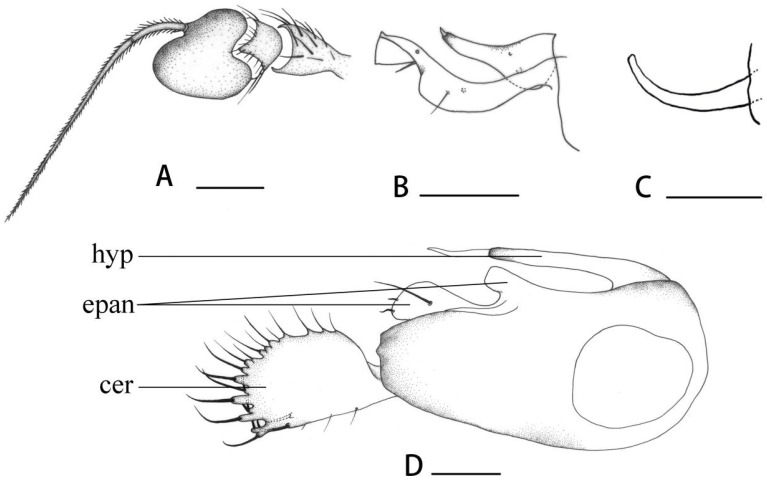
(**A**–**D**) *Dolichopus apicimaculatus* sp. nov., male. (**A**) antenna, lateral view (**B**) surstylus, lateral view (**C**) postgonite, lateral view (**D**) genitalia, lateral view. Abbreviations: hyp = hypandrium, epan = epandrial lobe, cer = male cercus. Scale bars: 0.1 mm.

Figure 2B, Figure 3I and Figure 4A–D

**Diagnosis.** Postpedicel short and blunt, with narrow baso-ventral area yellow; arista nearly apical. Wing slightly long and narrow, distinctly dark brown at tip; costal callus indistinct; M weakly bent. Calypter with black hairs. Hypandrium tubular at tip.

**Description. Male** (Figure 2B). Body length 3.7–3.8 mm, wing length 3.4–3.6 mm.

**Head** metallic green with pale grey pollinosity. Face yellow, almost as wide as postpedicel. Hairs and bristles on head black, but middle and lower postocular bristles yellow. Antenna (Figure 4A) with scape and pedicel yellow; postpedicel black except narrow baso-ventral area yellow, relatively small, almost as long as wide, blunt at tip; arista black with short hairs, basal segment 0.3 times as long as apical segment. Proboscis brown with black hairs; palpus yellow with black hairs and 1 black apical bristle.

**Thorax** metallic green with pale grey pollinosity. Hairs and bristles on thorax black; 5–6 irregularly biseriate acr short hair-like, 6 long strong dc. Scutellum with 2 pairs of sc and several short yellow marginal hairs, basal pair hair-like. Propleuron with short yellowish hairs and 1 black bristle on lower portion.

**Legs** entirely yellow. Fore coxa yellow; mid and hind coxae black, but yellow at tip; tarsi dark brown from tip of tarsomere 1 onwards. Hairs and bristles on legs black. Mid and hind coxae each with 1 outer bristle; mid and hind femora each with 1 preapical bristle. Fore tibia with 2–3 ad, 2 pd, 1 postero-ventral bristle, 2 black black apical bristles and 1 brown apico-ventral bristle (1/2 as long as fore tarsomere 1); mid tibia with 2–3 ad, 1 pd, 1 av and 4 apical bristles; hind tibia with 4 ad, 4 pd, 1 apico-dorsal bristle, 1 av and 2 apical bristles. Hind tarsomere 1 with 1 long ad and 1 long pd. Relative lengths of tibia and 5 tarsomeres of legs LI: 2.2:1.0:0.5:0.35:0.25:0.25; LII: 2.9:1.35:0.8:0.45:0.35:0.35; LIII: 2.9:1.2:1.0:0.7:0.45:0.4. **Wing** (Figure 3I) hyaline, tinged dark brown pattern on anterior part; veins black; costal callus indistinct; M weakly bent without rudimentary M_2_; CuAx ratio 0.6. Squama yellow with black hairs. Halter yellow.

**Abdomen** metallic green with pale grey pollinosity. Hairs and bristles on abdomen black. Male genitalia (Figure 4D): Epandrium distinctly longer than wide; inner epandrial lobe relatively short and thick, bent forwards, outer epandrial lobe large and wide with 2 curved apical bristles. Postgonite (Figure 4C) almost as long as dorsal lobe of surstylus. Male cercus nearly square with distinct finger-like marginal processes bearing apical bristles. Hypandrium somewhat tubular at tip.

**Female.** Unknown

**Type material. Holotype,** male, CHINA, Inner Mongolia, Hinggan, Tumuji, 2014.VII.24, Yanan Lv (CAU). **Paratype:** 1 male, CHINA, Inner Mongolia, Hulun Buir, Nuohanmenbu, 2014.VII.16, Yanan Lv (CAU); 1 male, CHINA, Inner Mongolia, Xilin Gol, Bieligutai, 2014.VII.12, Yanan Lv (CAU); 1 male, CHINA, Inner Mongolia, Mount Helan, Taerlingshuiku, 2010.VIII.11, Weina Cui (CAU).

**Distribution.** China (Inner Mongolia).

**Remarks.** This new species is somewhat similar to *Dolichopus zernyi* Parent, 1927, but can be distinguished by the postpedicel blunt at tip, 6 dc and the inner epandrial lobe narrow and curved at tip. In *D. zernyi*, the postpedicel is acute at tip, 5 dc are present, and the inner epandrial lobe is wide and not curved at tip [5]. This new species is somewhat similar to *Dolichopus asiaticus* Negrobov, 1973 but can be distinguished by the postpedicel blunt at tip and the male cercus with 4 distinct finger-like marginal processes and the ventral lobe of surstylus relatively thin and long and the phallus relatively long. In *D. asiaticus*, the postpedicel is pointed at tip and the male cercus is with indistinct finger-like marginal processes and the ventral lobe of surstylus is relatively short and strong and the phallus is very short [18,19].

**Etymology.** This species is named after the dark brown pattern on anterior part of wing.

#### 3.1.3. *Dolichopus aubertini* Parent, 1936

Figure 2C

*Dolichopus aubertini* Parent, 1936: 126. Type locality: China: “Tien-tsin” [=Tianjin] [20].

*Dolichopus aubertini* Parent, 1936. Yang et al. 2010: 492 [5].

**Diagnosis.** Postpedicel slightly short, blunt at tip, black with basal ventral area dark yellow. Fore tibia with 1 short apico-ventral bristle. Wing brown at tip; M bent in a right angle with rudimentary M_2_. Calypter with yellow hairs. Hypandrium long and thin with subapical denticles.

**Specimens examined.** 1 male, CHINA, Inner Mongolia, Mount Helan, Taerlingshuiku, 1900m, 2010.VIII.11, Wangli Hua (CAU); 1 male, CHINA, Inner Mongolia, Hulun Buir, Hailaerdaqiao, 2014.VII.19, Yanan Lv (CAU).

**Distribution.** China (Inner Mongolia, Hebei, Beijing, Tianjin).

#### 3.1.4. *Dolichopus bigeniculatus* Parent, 1926

Figure 2D and Figure 3H

*Dolichopus bigeniculatus* Parent, 1926: 114. Type locality: China: Shanghai, “Zi-Ka-Wei” [=Xujiahui] [21].

*Dolichopus bigeniculatus* Parent, 1926. Yang et al. 2010: 495 [5].

**Diagnosis.** Postpedicel with baso-ventral area dark yellow, 1.3 times longer than wide. Fore tibia brown with 1 apico-ventral bristle. M bent in a right angle; costal callus weak, stigma-like (Figure 3H). Epandrium with inner epandrial lobe finger-like; hypandrium sharp and curved at tip.

**Specimens examined.** 2 males 1 female, CHINA, Inner Mongolia, Chifeng, Daqinggou, 180m, 2014.VII.23, Ning Wang & Ding Yang (CAU); 1 male, CHINA, Inner Mongolia, Chifeng, Daqinggou, 2015.VII.8, Chifei Tang (CAU).

**Distribution.** China (Inner Mongolia, Beijing, Henan, Shandong, Shanxi, Sichuan, Anhui, Jiangsu, Zhejiang).

#### 3.1.5. *Dolichopus clavipes* Haliday, 1832

Figure 2E

*Dolichopus clavipes* Haliday, 1832: 365. Type locality: Ireland: Holywood [22].

*Dolichopus fuscipes* Haliday, 1832: 365. Type locality: Ireland: Holywood [23].

*Dolichopus trochanterarus* Zetterstedt, 1843: 529. Type locality: “Scandinaviae” [22].

*Dolichopus clavipes* Haliday, 1832. Yang et al. 2010: 502 [5].

**Diagnosis.** Antenna entirely black except scape dark yellow ventrally; postpedicel 1.25 times longer than wide. All femora brown dorsally; hind tibia distinctly thickened; hind femur with 7–11 yellow ventral bristles arranged in line (almost as long as femur thickness).

**Specimens examined.** 1 male, CHINA, Inner Mongolia, Mount Helan, Yaobayikenggou, 1900m, 2010.VIII.13, Lihua Wang (CAU); 12 males 6 females, CHINA, Inner Mongolia, Xilin Gol, Dongwuzhumuqin, 2014.VII.14, Yanan Lv (CAU); 2 males 1 female, CHINA, Inner Mongolia, Xilin Gol, Xiwuqi, 2014.VII.13, Yanan Lv (CAU).

**Distribution.** China (Inner Mongolia, Xinjiang).

#### 3.1.6. *Dolichopus galeatus* Loew, 1871

Figure 2F and Figure 3A

*Dolichopus galeatus* Loew, 1871: 271. Type locality: Russia: “Sibirien” [24].

*Dolichopus galeatus* Loew, 1871. Yang et al. 2010: 509 [5].

**Diagnosis.** Large sized. Hind femur with short dense ventral hairs. Fore tarsomere 1 slightly thickened, with short dense ventral and dorsal hairs, bristle-like; tarsomere 4 short, tarsomere 5 distinctly flattened. Male cercus nearly knife-like, distinctly longer than wide.

**Specimens examined.** 10 males 14 females, CHINA, Inner Mongolia, Arxan, Wuliquan, 1035m, 2014.VII.26, Ning Wang & Ding Yang (CAU).

**Distribution.** China (Inner Mongolia, Heilongjiang).

#### 3.1.7. *Dolichopus geniculatus* Stannius, 1831

Figure 2G

*Dolichopus geniculatus* Stannius, 1831: 135. Type locality: Germany: Hamburg [25].

*Dolichopus discrepans* Parent, 1928: 33. Type locality: Germany: “Allemagne” [26].

*Dolichopus geniculatus* Stannius, 1831. Yang et al. 2010: 510 [5].

**Diagnosis.** Postocular bristles entirely black. Antenna entirely black; postpedicel 1.2 times longer than wide. Legs black, tip of femora dark yellow, fore and mid tibia yellow. Hind femur with 12 black ventral bristles in a line. Costal callus weak stigma-like.

**Specimens examined.** 1 male, CHINA, Inner Mongolia, Mount Helan, Halawubeigou, 2010.VII.28, Weina Cui (CAU); 1 male, CHINA, Inner Mongolia, Mount Helan, Halawubeigou, 2010.VII.27, Lihua Wang (CAU); 1 male, CHINA, Inner Mongolia, Mount Helan, Halawubeigou, 2010.VII.28, Yan Li (CAU).

**Distribution.** China (Inner Mongolia, Jilin).

#### 3.1.8. *Dolichopus hilaris* Loew, 1862

Figure 2H

*Dolichopus hilaris* Loew, 1862: 297. Type locality: Poland: Miedzyrecz [27].

*Dolichopus hilaris* Loew, 1862. Yang et al. 2010: 517 [5].

**Diagnosis.** Fore tibia without apico-ventral bristle; 3/5 of basal hind femur with 10–12 brown ventral bristles, needle-like, shorter than thickness of femur. Costal callus relatively long and strong. Squama with brownish yellow hairs.

**Specimens examined.** 1 male, CHINA, Inner Mongolia, Hulun Buir, Nuomenhanbu, 2014.VII.16, Yanan Lv (CAU).

**Distribution.** China (Inner Mongolia, Xinjiang, Heilongjiang).

#### 3.1.9. *Dolichopus jiufengensis* sp. nov.

**Figure 5:**
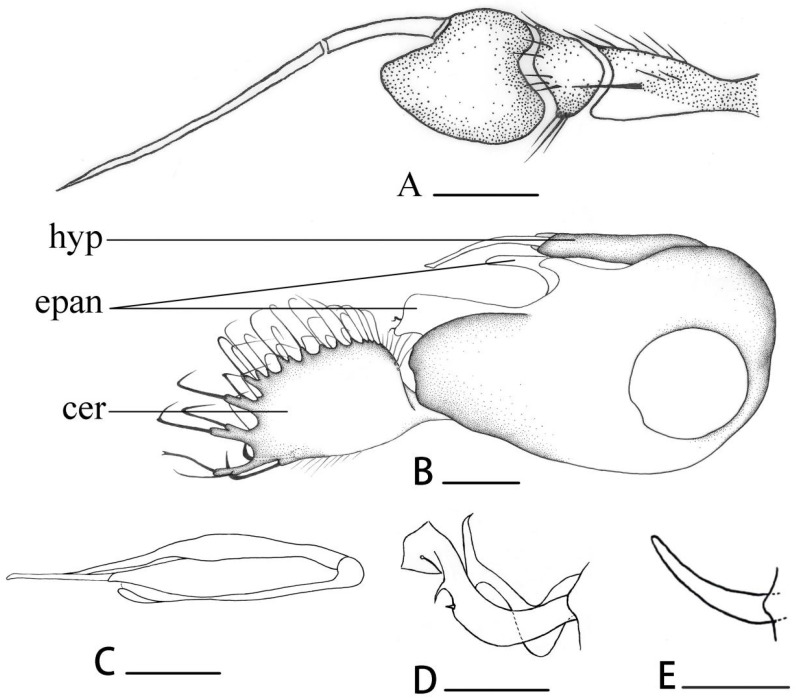
(**A**–**E**) *Dolichopus jiufengensis* sp. nov., male. (**A**) antenna, lateral view (**B**) genitalia, lateral view (**C**) hypandrium, ventral view (**D**) surstylus, lateral view (**E**) postgonite, lateral view. Abbreviations: hyp = hypandrium, epan = epandrial lobe, cer = male cercus. Scale bars: 0.1 mm.

Figure 2I, Figure 3G and Figure 5A–E

**Diagnosis.** Postpedicel entirely black. Costal callus long and thick; M bent in a right angle, with rudimentary M_2_. Fore tibia with long ventral bristles apically (half as long as fore tarsomere 1); hind tarsomere 1 with 2 dorsal bristles. Hypandrium obtuse apically; outer epandrial lobe long and thin.

**Description. Male** (Figure 2I). Body length 4.0–4.5 mm, wing length 4.5–4.9 mm.

**Head** metallic green with pale grey pollinosity. Face with silvery pollinosity, almost as wide as postpedicel. Hairs and bristles on head black, but middle and lower postocular bristles and posteroventral hairs yellow. Antenna (Figure 5A) blackish, but scape dark brownish yellow at basal half of ventral surface; postpedicel black, 1.4 times longer than wide, blunt at tip; arista black with short hairs, basal segment 0.35 times as long as apical segment. Proboscis brownish yellow with brown hairs; palpus yellow with blackish hairs and 1 black apical bristle.

**Thorax** metallic green with pale grey pollinosity. Hairs and bristles on thorax black; 7–8 irregularly biseriate acr short hair-like, 6 long strong dc. Scutellum with 2 pairs of sc and several short marginal yellow hairs, basal pair hair-like. Propleuron with short yellow hairs and 1 black bristle on lower portion.

**Legs** mostly yellow. Fore coxa yellow; mid coxa black, yellow at tip; hind coxa yellow, tinged with blackish speckle at base. Fore and mid tarsi brownish to brown from tip of tarsomere 1 onwards; tip of tibia and tarsus black. Hairs and bristles on legs black. Mid and hind coxae each with 1 outer bristle; mid and hind femora each with 1 preapical bristle. Fore tibia with 2 ad, 3 pd, 1 postero-ventral bristle, 2 apical bristles and 1 brown apico-ventral bristle at tip (half as long as fore tarsomere 1); mid tibia with 4 ad, 2 pd, 1 av and 4 apical bristles; hind tibia with 4 ad, 4 pd, 1 apico-dorsal bristle, 1 av and 2 apical bristles. Fore tarsomere 1 with 1 short brown ventral bristle at base; hind tarsomere 1 with 2 long dorsal bristles. Relative lengths of tibia and 5 tarsomeres of legs LI: 2.8:1.2:0.6:0.5:0.35:0.3; LII: 3.8:2.0:1.05:0.8:0.5:0.4; LIII: 4.0:1.8:1.8:0.6:0.5:0.25. **Wing** (Figure 3G) hyaline, slightly tinged greyish; veins blackish; costal callus long and thick; M bent in a right angle with rudimentary M_2_; CuAx ratio 0.65. Squama yellow with black hairs. Halter yellow.

**Abdomen** metallic green with pale grey pollinosity. Hairs and bristles on abdomen black. Sternites 2–3 with dark yellow hairs. Male genitalia (Figure 5B): Epandrium distinctly longer than wide; inner epandrial lobe relatively short and narrow but long and thin on the opposite, finger-like, outer epandrial lobe wide and large with 1 winding apical bristle. Postgonite (Figure 5E) shorter than dorsal lobe of surstylus. Male cercus nearly square with distinct finger-like marginal processes bearing apical bristles. Hypandrium (Figure 5C) relatively thick.

**Female.** Body length 4.2–5.1 mm, wing length 4.5–4.8 mm. Similar to male but wing without costal callus.

**Type material. Holotype,** male, CHINA: Inner Mongolia, Mount Jiufeng, Erdaogou, 1200–1500m, 2013.VIII.3, Xiumei Lu (CAU). **Paratypes:** 4 males 6 females, same data as holotype (CAU); 1 male 1 female, CHINA, Inner Mongolia, Mount Jiufeng, Erdaogou, 1200–1500m, 2013.VIII.3, Xiao Zhang (CAU); 1 male, CHINA, Inner Mongolia, Mount Jiufeng, Toudaogou, 1500–1600m, 2013.VIII.4, Xiao Zhang (CAU); 2 males 2 females, CHINA, Inner Mongolia, 2013.VII.22, Xiumei Lu (CAU); 1 male 1 female, CHINA, Inner Mongolia, Xilin Gol, Dongwuzhumuqin, 2014.VII.14, Chifeng, Saihanwula, 1070m, 20114, Yanan Lv (CAU).

**Distribution.** China (Inner Mongolia).

**Remarks.** This new species is somewhat similar to *Dolichopus cuneipennis* Parent, 1926, but can be distinguished from the latter by the postpedicel 1.4 times longer than wide and entirely black and hind tibia black at tip. In *D. cuneipennis*, the postpedicel is 1.1 times longer than wide, its baso-ventral area is brownish yellow, and the apical 2/3 of the hind tibia is black [5]. This new species is somewhat similar to *Dolichopus stackelbergi* Smirnov, 1948 but can be distinguished by the face as wide as postpedicel and the hind tibia normal and the male cercus nearly square and the squama with black hairs. In *D. stackelbergi*, the face is twice as wide as postpedicel and the hind tibia is somewhat thickened and the male cercus is narrow at base, nearly trapezoidal and the squama is with dominance of black hairs and some yellow hairs [18,28].

**Etymology.** The species is named after the collecting area, Mount Jiufeng.

#### 3.1.10. *Dolichopus linearis* Meigen, 1824

Figure 2J and Figure 3C

*Dolichopus linearis* Meigen, 1824: 84. Type locality: not given [17].

*Dolichopus plebeius* Meigen, 1824: 99. Type locality: England [17].

*Dolichopus parvulus* Zetterstedt, 1843: 555. Type locality: Scania meridionali, Lund; Ostrogothia, Wadstena; Hamburgum [Sweden, Germany] [29].

*Dolichopus agilis* Zetterstedt, 1849: 3081 [30].

*Dolichopus linearis* Meigen, 1824. Yang et al. 2010: 526 [5].

**Diagnosis.** Antenna yellow but scape and pedicel brown ventrally; postpedicel black, 1.2 times longer than wide, sharp at tip. Costal callus long and thick (Figure 3C); M weakly bent. Coxae yellow, only mid coxa with 1 black fleck. Fore tibia with 1 long thin brown apico-ventral bristle. Epandrial inner lobe long and strong.

**Specimens examined.** 8 males, CHINA, Inner Mongolia, Chifeng, Saihanwula, 2013.VII.22, Xiumei Lu (CAU); 2 males 3 females, CHINA, Inner Mongolia, Mount Jiufeng, Erdaogou, 1400–1500m, 2013.VIII.3, Xiao Zhang (CAU); 8 males 8 females, CHINA, Inner Mongolia, Mount Jiufeng, Toudaogou, 1500–1600m, 2013.VIII.4~5, Xiao Zhang (CAU); 17 males 13 females, CHINA, Inner Mongolia, Mount Helan, Xiangchizigou, 1900m, 2013.VII.30, Xiao Zhang (CAU); 1 male 1 female, CHINA, Inner Mongolia, Arxan, Wuliquan, 1035m, 2014.VII.26, Ning Wang & Ding Yang (CAU).

**Distribution.** China (Inner Mongolia, Heilongjiang, Jilin, Beijing, Gansu, Xinjiang, Qinghai).

#### 3.1.11. *Dolichopus luae* sp. nov.

**Figure 6:**
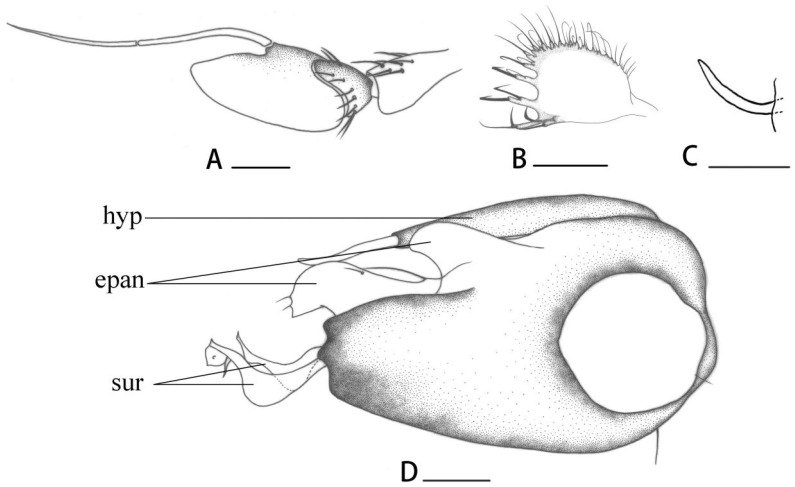
(**A**–**D**) *Dolichopus luae* sp. nov., male. (**A**) antenna, lateral view (**B**) male cercus, lateral view (**C**) postgonite, lateral view (**D**) genitalia, lateral view. Abbreviations: hyp = hypandrium, epan = epandrial lobe, sur = surstylus. Scale bars: 0.1 mm.

Figure 2K and Figure 6A–D

**Diagnosis.** Antenna blackish but scape and pedicel dark yellow ventrally, postpedicel ventrally dark yellow at base; postpedicel moderately elongated, 2 times longer than wide, blunt at tip. Costal callus long and thick; M weakly bent without rudimentary M_2_. Fore tibia with relatively long ventral bristles apically (half as long as fore tarsomere 1); hind tarsomesre 1 with 1 dorsal bristle.

**Description. Male** (Figure 2K). Body length 3.8–3.9 mm, wing length 3.4–3.5 mm.

**Head** metallic green with pale grey pollinosity. Face with silvery pollinosity, almost as wide as postpedicel. Hairs and bristles on head black, but middle and lower postocular bristles and posteroventral hairs yellow. Antenna (Figure 6A) blackish but scape, pedicel dark yellow ventrally, postpedicel ventrally dark yellow at base; postpedicel moderately elongated, 2 times longer than wide, blunt at tip; arista blackish with short hairs, basal segment 0.85 times as long as apical segment. Proboscis brownish yellow with brown hairs; palpus yellow with brown hairs and 1 brown apical bristle.

**Thorax** metallic green with pale grey pollinosity. Hairs and bristles on thorax black; 6–7 irregularly biseriate acr short hair-like, 6 long strong dc. Scutellum with 2 pairs of sc and several short marginal yellow hairs, basal pair hair-like. Propleuron with short yellow hairs and 1 black bristle on lower portion.

**Legs** entirely yellow. Fore coxa yellow; mid and hind coxae blackish, but yellow at apical margin; fore and mid tarsi brownish to brown from tip of tarsomere 1 onwards; hind tibia and tarsus black. Hairs and bristles on legs black; mid and hind coxae each with 1 outer bristle; mid and hind femora each with 1 preapical bristle. Fore tibia with 1–2 ad, 2 pd, 2 apical bristles and 1 brown apico-ventral bristle (half as long as fore tarsomere 1); mid tibia with 3 ad, 2 pd, 1 av and 4 apical bristles; hind tibia with 4 ad, 4 pd, 1 apico-dorsal bristle, 1 av and 2 apical bristles. Fore tarsomere 1 with 1 short brown ventral bristle at base; hind tarsomere 1 with 1 long dorsal bristle and 1 short av. Relative lengths of tibia and 5 tarsomeres of legs LI: 2.1:1.0:0.5:0.4:0.3:0.25; LII: 2.9:1.5:0.8:0.7:0.45:0.4; LIII: 3.2:1.3:1.4:0.9:0.6:0.45. **Wing** hyaline; veins blackish; costal callus long and thick; M weakly bent without rudimentary M_2_; CuAx ratio 0.55. Squama yellow with blackish hairs. Halter yellow.

**Abdomen** metallic green with pale grey pollinosity. Hairs and bristles on abdomen black. Male genitalia (Figure 6D): Epandrium distinctly longer than wide; inner epandrial lobe relatively small, outer epandrial lobe wide and large with 2 apical bristles (one strong and straight, one thin and bent), sunken areas between two lobes relatively small. Postgonite (Figure 6C) shorter than dorsal lobe of surstylus. Male cercus (Figure 6B) nearly square with distinct finger-like marginal processes bearing apical bristles. Hypandrium somewhat thick at tip.

**Female.** Unknown

**Type material. Holotype,** male, CHINA: Inner Mongolia, Saihanwula, Dadonggou, 1200m, 2013.VII.25, Xiumei Lu (CAU). **Paratypes:** 1 male, same data as holotype (CAU).

**Distribution.** China (Inner Mongolia).

**Remarks.** This new species is somewhat similar to *Dolichopus longicornis* Stannius, 1831, but can be distinguished from the latter by the following features: antenna moderately elongated, postpedicel 2 times longer than wide and blunt at tip; scape and pedicel dark yellow ventrally; postpedicel ventrally dark yellow at base; hind tarsomere 1 with 1 dorsal bristle. In *D. longicornis*, the antenna is distinctly elongated, the postpedicel is 2.6 times longer than wide and blunt at tip, the antenna is entirely black and the hind tarsomere 1 is with 2 dorsal bristles [5]. This new species is somewhat similar to *Dolichopus albipalpus* Negrobov, 1973 but can be distinguished from the face as wide as postpedicel and the fore tibia with no antero-dorsal bristle in apical part and the outer epandrial lobe with 1 short bristle at base and 1 long bristle between outer epandrial lobe and inner epandrial lobe and the ventral lobe of surstylus with 1 bristle and the dorsal lobe of surstylus with no bristle. In *D. albipalpus*, the face is narrower than the postpedicel and the fore tibia is with a row of short antero-dorsal bristles in apical part and the outer epandrial lobe is with 1 relatively long bristle at base and 1 relatively short bristle between outer epandrial lobe and inner epandrial lobe and the ventral lobe of surstylus is with more than 1 bristle and the dorsal lobe of surstylus is with several spine-like bristles [18,19].

**Etymology.** The species is named after the collector Xiumei Lu.

#### 3.1.12. *Dolichopus longicornis* Stannius, 1831

**Figure 7:**
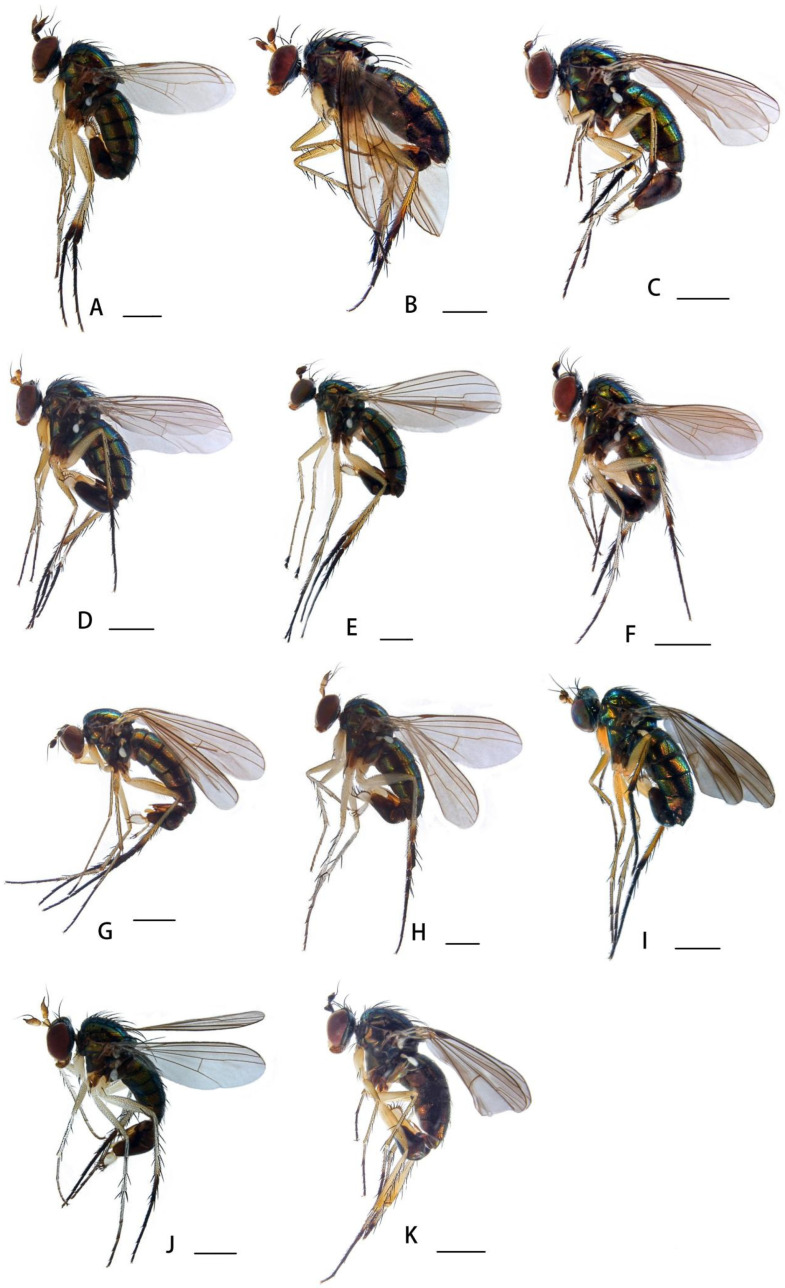
(**A**–**K**) Habitus, lateral view. (**A**) *Dolichopus (D.) longicornis* Stannius, 1831, male (**B**) *Dolichopus (D.) martynovi* Stackelberg, 1930, male (**C**) *Dolichopus (D.) orientalis* Parent, 1927, male (**D**) *Dolichopus (D.) plumipes* Scopoli, 1763, male (**E**) *Dolichopus (D.) plumitarsis* Fallén, 1823, male (**F**) *Dolichopus (D.) ringdahli* Stackelberg, 1929, male (**G**) *Dolichopus (D.) simius* Parent, 1927, male (**H**) *Dolichopus (D.) tewoensis* Yang, 1998, male (**I**) *Dolichopus (D.) zernyi* Parent, 1927, male (**J**) *Dolichopus (D.) zhoui* Zhang, Yang *et* Grootaert, 2004, male (**K**) *Dolichopus (H.) rotundipennis* Loew, 1848. Scale bars: 1 mm.

Figure 3D and Figure 7A

*Dolichopus longicornis* Stannius, 1831: 53. Type locality: not given [Germany: Hamburg, Breslau] [25].

*Dolichopus longicornis* Stannius, 1831. Yang et al. 2010: 528 [5].

**Diagnosis.** Antenna entirely black; postpedicel distinctly elongated; 2.3 times longer than wide, blunt at tip. Mid and hind femora black, but yellow at base; fore tibia with 1 long apico-ventral bristle; hind tarsomere 1 with 1 dorsal bristle. Costal callus long and strong.

**Specimens examined.** 62 males 20 females, CHINA, Inner Mongolia, Mount Helan, Xiangchizigou, 1900m, 2013.VII.30, Xiao Zhang (CAU); 2 males 2 females, CHINA, Inner Mongolia, Mount Helan, Yaobayikenggou, 1900m, 2010.VIII.13, Lihua Wang (CAU); 1 male, CHINA, Inner Mongolia, Hinggan, Tumuji, Paozi, 2014.VII.23, Yanan Lv (CAU); 12 males 5 females, CHINA, Inner Mongolia, Xinlin Gol, Dongwuqi, 2014.VII.14, Yanan Lv (CAU).

**Distribution.** China (Inner Mongolia, Shanxi).

#### 3.1.13. *Dolichopus longipilosus* Zhang *et* Yang, 2008

*Dolichopus (Dolichopus) longipilosus* Zhang *et* Yang, 2008: 2523. Type locality: China: Inner Mongolia (Tuweibashan) [16].

*Dolichopus (Dolichopus) longipilosus* Zhang *et* Yang, 2008. Yang et al. 2010: 529 [5].

**Diagnosis.** Hind femur with 4–5 long yellow ventral hairs (distinctly longer than thickness of femur).

**Specimens examined.** 1 male, CHINA, Inner Mongolia, Hulun Buir, 1975.VII.8 (CAU).

**Distribution.** China (Inner Mongolia).

#### 3.1.14. *Dolichopus martynovi* Stackelberg, 1930

Figure 3B,E and Figure 7B

*Dolichopus martynovi* Stackelberg, 1930: 145. Type locality: Russia: “Siberiae orientalis prov. Austro-Ussuriensis propre pagum Tigrovaja, distr. Sutshanicus; litus meridionalis laci Chanka promotorium Rjabokonj; prope pagum Staraja Devitza, pagum Kamenj-Rybolov; Vladivostok” [31].

*Dolichopus martynovi* Stackelberg, 1930. Yang et al. 2010: 535 [5].

**Diagnosis.** Postpedicel entirely black. Apical half of hind tibia black; apical half of hind femur with 5~6 pale brown long ventral bristles, nearly as long as femur thickness (Figure 3B). Fore tibia without apico-ventral bristles. Costal callus stigma-like; M bent in a right angle with rudimentary M_2_ (Figure 3E).

**Specimens examined.** 2 males, CHINA, Inner Mongolia, Mount Jiufeng, Erdaogou, 1400–1500m, 2013.VIII.3, Xiao Zhang (CAU); 3 males 7 females, CHINA, Inner Mongolia, Keerqin, 460m, 2008.VII.19, Gang Yao (CAU); 16 males 19 females, CHINA, Inner Mongolia, Xinganmeng, Tumuji, 2014.VII.23, Yanan Lv (CAU).

**Distribution.** China (Inner Mongolia, Heilongjiang, Jilin, Hebei, Ningxia, Shanxi, Xinjiang).

#### 3.1.15. *Dolichopus orientalis* Parent, 1927

Figure 3F and Figure 7C

*Dolichopus orientalis* Parent, 1927: 463. Type locality: China: Mandchourie: Ourga à Tsitsikhar [32].

*Dolichopus orientalis* Parent, 1927. Yang et al. 2010: 543 [5].

**Diagnosis.** Antenna yellow; scape brown dorsally; postpedicel entirely black, 1.3 times longer than wide. Half hind femur with sevral ventral yellow hairs, slightly shorter than thickness of femur. Costal callus stigma-like (Figure 3F). Clapyter with yellow hairs.

**Specimens examined.** 1 male, CHINA, Inner Mongolia, Hulun Buir, Hailaerdaqiao, 2014.VII.19, Yanan Lv (CAU); 1 male, CHINA, Inner Mongolia, Chifeng, Saihanwula, 1200m, 2013.VII.24, Xiumei Lu (CAU).

**Distribution.** China (Inner Mongolia, Heilongjiang).

#### 3.1.16. *Dolichopus plumipes* Scopoli, 1763

Figure 7D

*Musca plumipes* Scopoli, 1763: 334. Type locality: “carnioliae indigena” [33].

*Dolichopus pennitarsis* Fallén, 1823: 11. Type locality: Sweden: “Ostrogothia et Scania” [34].

*Dolichopus ciliatus* Walker, 1849: 661. Type locality: Canada: Ontario, Hudson’s Bay, Albany River, St. Martin’s Falls [35].

*Dolichopus sequax* Walker, 1849. List Dipt. Brit. Mus. 3: 666. Type locality: Canada: Ontario, Hudson’s Bay, Albany River, St. Martin’s Falls [35].

*Dolichopus nigroapicalis* Van Duzee, 1930: 125. Type locality: USA: Colorado, Longs Peak Inn [36].

*Dolichopus plumipes* (Scopoli): Negrobov, 1991: 111 [37].

*Musca plumipes* Scopoli, 1763. Yang et al. 2010: 544 [5].

**Diagnosis.** Antenna: scape and pedicel dark yellow, postpedicel black with narrow ventral area yellow at base. Mid tibia distinctly narrowed with brown strips dorsally; mid tarsomere 1 with short marginal feather-like bristles.

**Specimens examined.** 2 males, CHINA, Inner Mongolia, Mount Jiufeng, Erdaogou, 1400–1500m, 2013.VIII.3, Xiumei Lu (CAU); 1 male 1 female, CHINA, Inner Mongolia, Arxan, Wuliquan, 1035m, 2013.VII.26, Ning Wang & Ding Yang (CAU).

**Distribution.** China (Inner Mongolia, Heilongjiang, Hebei, Henan, Shanxi, Xinjiang, Qinghai, Tibet).

#### 3.1.17. *Dolichopus plumitarsis* Fallén, 1823

Figure 7E

*Dolichopus plumitarsis* Fallén, 1823:10. Type locality: not given (Sweden) [34].

*Dolichopus plumitarsis* Fallén, 1823. Yang et al. 2010:546 [5].

**Diagnosis.** Antenna black but scape dark brown ventrally. Fore tarsomeres 4–5 black, distinctly flattened with feather-like dorsal hairs; tarsomere 4 1.5–2 times longer than wide. Costal callus indistinct; M weakly bent with or without rudimentary M_2_. Male cercus nearly long knife-like.

**Specimens examined.** 2 males, CHINA, Inner Mongolia, Mount Jiufeng, Erdaogou, 1400–1500m, 2013.VIII.3, Xiao Zhang (CAU); 3 males 2 females, CHINA, Inner Mongolia, Mount Helan, Nansi, 3200–3300m, 2013.VII.31, Xiao Zhang (CAU); 1 male, CHINA, Inner Mongolia, Arxan, Wuliquan, 1035m, 2013.VII.26, Ning Wang & Ding Yang (CAU).

**Distribution.** China (Inner Mongolia, Heilongjiang, Hebei, Beijing, Xinjiang).

#### 3.1.18. *Dolichopus ringdahli* Stackelberg, 1929

Figure 7F

*Dolichopus ringdahli* Stackelberg, 1929: 160. Type locality: Russia: “Kreis Jakutsk: Keedej-See; Süd-Ussuri-Gebiet: Tigrovaya. Kreis Sutshan” [31].

*Dolichopus ringdahli* Stackelberg, 1929. Yang et al. 2010: 551 [5].

**Diagnosis.** Antenna entirely black; postpedicel short, 1.1 times longer than wide. Fore coxa black at base; fore tibia without apico-ventral bristle. Costal callus indistinct.

**Specimens examined.** 3 males, CHINA, Inner Mongolia, Tongliao, Daqinggou, 200–300m, 2013.VII.18, Xiao Zhang (CAU); 9 males 15 females, CHINA, Inner Mongolia, Arxan, Wuliquan, 1035m, 2014.VII.26, Ning Wang (CAU); 13 males 9 females, CHINA, Inner Mongolia, Tongliao, Daqinggou, 180m, 2014.VII.22, Ning Wang & Ding Yang (CAU).

**Distribution.** China (Inner Mongolia, Jilin).

#### 3.1.19. *Dolichopus simius* Parent, 1927

Figure 7G

*Dolichopus simius* Parent, 1927: 465. Type locality: Russia: Sibérie: environs d’Irkutsk [32].

*Dolichopus simius* Parent, 1927. Yang et al. 2010: 561 [5].

**Diagnosis.** Antenna entirely black; postpedicel short, 1.1 times longer than wide. Fore coxa black at base; fore tibia without apico-ventral bristle. Costal callus indistinct.

**Specimens examined.** 3 males, CHINA, Inner Mongolia, Tongliao, Daqinggou, 200–300m, 2013.VII.18, Xiao Zhang (CAU); 9 males 15 females, CHINA, Inner Mongolia, Arxan, Wuliquan, 1035m, 2014.VII.26, Ning Wang (CAU); 13 males 9 females, CHINA, Inner Mongolia, Tongliao, Daqinggou, 180m, 2014.VII.22, Ning Wang & Ding Yang (CAU).

**Distribution.** China (Inner Mongolia, Jilin).

#### 3.1.20. *Dolichopus tewoensis* Yang, 1998

Figure 7H

*Dolichopus tewoensis* Yang, 1998: 174. Type locality: Gansu (Tewo) [38].

*Dolichopus tewoensis* Yang, 1998. Yang et al. 2010: 566 [5].

**Diagnosis.** Postpedicel with ventral area dark yellow at base; arista at basal 1/3 of dorsal margin of postpedicel. Fore and hind coxae yellow; mid coxa black. Hind tarsomere 1 with 3 long dorsal bristle. Costal callus long and strong.

**Specimens examined.** 1 male 2 females, CHINA, Inner Mongolia, Mount Jiufeng, Erdaogou, 1400–1500m, 2013.VIII.3, Xiao Zhang (CAU); 2 males, CHINA, Inner Mongolia, Mount Jiufeng, Toudaogou, 1500–1600 m, 2010.VIII.4, Xiao Zhang (CAU).

**Distribution.** China (Inner Mongolia, Shanxi, Beijing, Gansu).

#### 3.1.21. *Dolichopus zernyi* Parent, 1927

Figure 7I

*Dolichopus zernyi* Parent, 1927: 52. Type locality: Russia: “Sarepta” [=Krasnoarmeysk, near Volgograd] [39].

*Dolichopus zernyi* Parent, 1927. Yang et al. 2010: 581 [5].

**Diagnosis.** Postpedicel with ventral area yellow basally, 1.1 times longer than wide. Wing grey at apical half above Ma; M-Cu tinged grey; costal callus stigma-like without rudimentary M_2_; calypter with part or entirely yellow hairs. Male cercus with short denticles.

**Specimens examined.** 1 male 4 females, CHINA, Inner Mongolia, Sonid Left Banner, Chaganaobao, 2014.VII.11, Yanan Lv (CAU).

**Distribution.** China (Inner Mongolia, Xinjiang).

#### 3.1.22. *Dolichopus zhoui* Zhang, Yang *et* Grootaert, 2004

Figure 7J

*Dolichopus zhoui* Zhang, Yang *et* Grootaert, 2004: 556. Type locality: China: Beijing, Mentougou [40].

*Dolichopus zhoui* Zhang, Yang *et* Grootaert, 2004. Yang et al. 2010: 585 [5].

**Diagnosis.** Antenna mostly yellow; postpedicel distinctly enlongated, 2.4 times longer than wide, sharp at tip; arista at basal 1/3 of postpedicel. Fore and hind coxae yellow; mid coxa black; fore tibia with 1 long apico-ventral bristle. Costal callus lacking; M weakly bent.

**Specimens examined.** 1 male 5 females, CHINA, Inner Mongolia, Tongliao, Daqinggou, 200–300m, 2013.VII.18, Xiao Zhang (CAU); 4 males 1 female, CHINA, Inner Mongolia, Tongliao, Daqinggou, 180m, 2013.VII.22, Ning Wang & Ding Yang (CAU); 1 male, CHINA, Inner Mongolia, Tongliao, Daqinggou, 180m, 2014.VII.24, Ning Wang & Ding Yang (CAU).

**Distribution.** China (Inner Mongolia, Beijing).

#### 3.1.23. *Dolichopus (Hygroceleuthus) rotundipennis* Loew, 1848

Figure 7K

*Dolichopus (Hygroceleuthus) rotundipennis* Loew, 1848: 329. Type locality: Russia: “Sibirien” [41].

Dolichopus (Hygroceleuthus) rotundipennis Loew, 1848. Yang et al. 2010: 590 [5].

**Diagnosis.** Antenna black, scape and pedicel distinctly elongated; postpedicel 1.6 times longer than wide, sharp at tip. Wing wide and large, hind margin of basal half with 1 distinct emargination; costal callus long but strong; M without rudimentary M_2_; CuAx ratio 1. Calypter with yellow hairs.

**Specimens examined.** 2 males 1 female, CHINA, Inner Mongolia, Xilin Gol, Xiwuqi, 2014.VII.13, Yannan Lv (CAU); 1 male, CHINA, Inner Mongolia, Hulun Buir, Hailaerdaqiao, 2014.VII.19, Yanan Lv (CAU); 1 male 3 females, CHINA, Inner Mongolia, Hulun Buir, Nuomenhanbu, 2014.VII.16, Yanan Lv (CAU).

**Distribution.** China (Inner Mongolia, Qinghai).

### 3.2. Key to the Species of Dolichopus from Inner Mongolia

1. Clypeus not reaching lower eye margin; scape and pedicel not elongated.................2

- Clypeus ending at lower eye margin; scape and pedicel distinctly elongated..............................................................................***D. (H.) rotundipennis* Loew**

2. Fore or mid tarsus flattened and plumate.........................................................................3

- All tarsi simple.......................................................................................................................6

3. Mid tarsomere 1 simple; fore tarsomeres 4–5 flattened with filamentous dorsal hairs.........................................................................................................................................4

- Mid tarsomere 1 with feather-like lateral bristles; fore tarsomeres 4–5 simple.....***D. (D.) plumipes* Scopoli**

4. Hind femur without group of ventral hair; aquama black haired................................5

- Hind femur with group of short hair; squama pale haired..................***D. (D.) galeatus* Loew**

5. Fore tarsomere 4 flattened, 1.5–2.0 times longer than wide......................***D. (D.) plumitarsis* Fallén**

- Fore tarsomere 4 relatively long, 2.5 times longer than wide.................***D. (D.) simius* Parent**

6. Femora blackish or mostly black (Figure 3A)...................................................................7

- Femora mostly or wholly yellow (Figure 3B,C).............................................................8

7. Postocullar bristles yellow; hind tibia distinctly thickened; hypandrium sharp at tip.................................................................................................***D. (D.) clavipes* Haliday**

- Postocullar bristles black; hind tibia normal; hypandrium blunt at tip..........................................................................................***D. (D.) geniculatus* Stannius**

8. Hind femur with (Figure 3B) long ventral hairs or bristles............................................9

- Hind femur without (Figure 3C) ventral hairs or bristles, at most with short hairs.......................................................................................................................................12

9. M weakly bent without rudimentary M_2_ (Figure 3D)....................................................10

- M strongly bent in a right angle with rudimentary M_2_ (Figure 3E).........................***D. (D.) martynovi* Stackelberg**

10. Costal callus long and strong (Figure 3D); squama yellow with black or brownish yellow hairs..........................................................................................................................11

- Costal callus stigma-like (Figure 3F); squama yellow with yellow hairs..............................................................................................***D. (D.) orientalis* Parent**

11. Hind tarsomere 1 with 2–3 dorsal bristle; postpedicel sharp at tip; hind femur with 10–11 brown bristles, shorter than thickness of femur.......................................***D. (D.) hilaris* Loew**

- Hind tarsomere 1 with 1 dorsal bristle; postpedicel sharp at tip; hind femur with 4–5 yellow long ventral hairs, longer than thickness of femur........................***D. (D.) longipilosus* Zhang et Yang**

12. Fore tibia with distinct apico-ventral bristles..................................................................13

- Fore tibia with indistinct or without apico-ventral bristle..............................................22

13. M strongly bent in a right angle with rudimentary M_2_....................................................14

- M weakly bent without rudimentary M_2_..........................................................................16

14. Wing hyaline or slightly greyish anteriorly......................................................................15

- Wing distinctly brown anteriorly.................................................***D. (D.) aubertini* Parent**

15. 15 Costal callus long and strong (Figure 3G); epandrial outer lobe with 1 winding bristle apically (Figure 5B); hypandrium relatively thick (Figure 5C)...........***D. (D.) jiufengensis* sp. nov.**

- Costal callus stigma-like (Figure 3H); epandrial outer lobe with 3 strong bristles apically; hypandrium curved at tip............................................***D. (D.) bigeniculatus* Parent**

16. Only fore coxa yellow, mid and hind coxae black or only mid coxa black...................17

- All coxae yellow, but mid coxa with a black spot...........................***D. (D.) linearis* Meigen**

17. Costal callus long and strong..............................................................................................18

- Costal callus indistinct or stigma-like................................................................................20

18. Hind coxa blackish, yellow at tip; hind tarsomere 1 with 1 long dorsal bristle and 1 apico-dorsal bristle................................................................................***D. (D.) luae* sp. nov.**

- Hind coxa yellow or mostly yellow with brown spot; hind tarsomere 1 with 2 dorsal bristles...................................................................................................................................19

19. Postpedicel 2.6 times longer than wide; hind coxa mostly yellow with brown spot; hind tarsomere 1 with no lateral bristles.............................***D. (D.) longicornis* Stannius**

- Postpedicel 2.2 times longer than wide; hind coxa yellow; hind tarsomere 1 with 1 lateral bristle.....................................................................................***D. (D.) tewoensis* Yang**

20. Costal callus indistinct (Figure 3I); squama black haired................................................21

- Costal callus stigma-like; squama pale haired.................................***D. (D.) zernyi* Parent**

21. Scape and pedicel yellow; postpedicel as long as wide, blunt at tip; arista basal segment 0.3 times as long as apical segment (Figure 4A)........................***D. (D.) apicimaculatus* sp. nov.**

- Scape and pedicel black dorsally; postpedicel 2.4 times longer than wide, sharp at tip; aristal basal segment 0.7 times as long as apical segment.....................................***D. (D.) zhoui* Zhang, Yang et Grootaert**

22. Scape yellow but brown dorsally, pedicel brownish; costal callus long and thick; hypandrium sharp at tip................................................................***D. (D.) agilis* Meigen**

- Scape and pedicel black; costal callus indistinct; hypandrium relatively curved at tip........................................................................................***D. (D.) ringdahli* Stackelberg**

### 3.3. Potential Geographic Distribution of Dolichopus in Inner Mongolia

**Figure 8:**
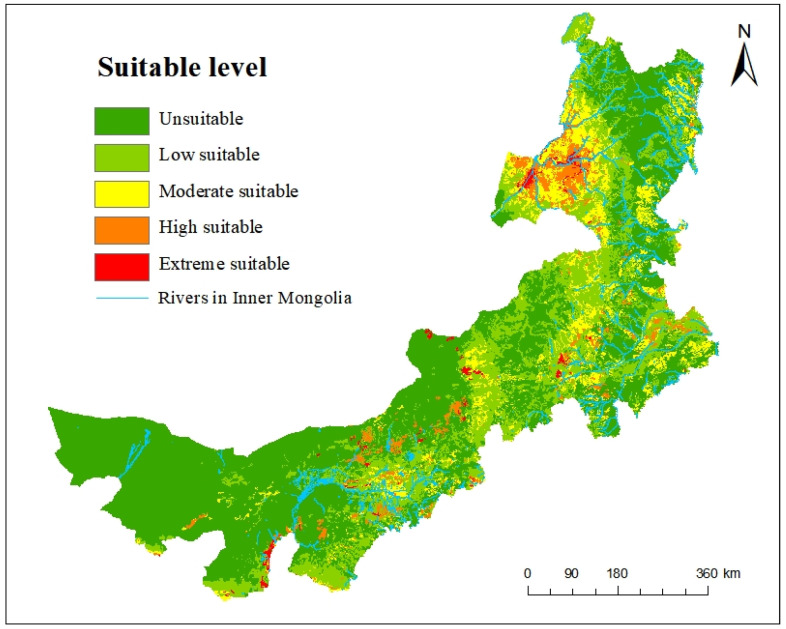
Potential geographic distribution of *Dolichopus* in Inner Mongolia.

The model projection results showed that the middle and northern parts of Inner Mongolia had a wide range of extremely, highly, and moderately suitable and unsuitable areas for *Dolichopus* with areas of 1.208729, 5.80061, 12.401, 32.4708 and 66.14889 km^2^, respectively. The moderately, extremely and highly suitable areas for *Dolichopus* are located in the Hailar River Basin, Argun River Basin, Nen River Basin, and Hulun Lake of northeastern Inner Mongolia; the upper and middle reaches of the Taoer River Basin, lower reaches of the Huolin River, Xiliao River Basin, Xar Moron River Basin, middle and southern part of the Da Hinggan Mountains of eastern Inner Mongolia; the Otindag Sandy Land, Daqing Mountain Range, eastern part of the Yellow River Basin in middle Inner Mongolia; and the Helan Mountain Ranges and Yabulai Mountain of western Inner Mongolia (Figure 8).

## 4. Discussion

So far, three subgenera, *Dolichopus*, *Hygroceleuthus* and *Macrodolichopus* are known to occur in China, and two of them are known to occur in Inner Mongolia. Brooks considered *Hygroceleuthus* and *Macrodolichopus* of no taxonomic value due to the variable characters of the antenna and clypeus [42]. However, Negrobov considered *Hygroceleuthus* distinct based on the antennae and wings of both the males and females [43]. *Hygroceleuthus* is considered by some scholars to be a subgenus while others considered it to be an independent genus or synonym [16,23,37,44,45,46,47,48]. It includes 13 species worldwide and 4 species in China [5,42,49]. *D.(H). Brevifacies* Stackelberg, 1925 and Tibet. *D.(H). rotundipennis* Loew, 1848 were distributed in Qinghai. *D.(H). latipennis* Fallén, 1832 and *D.(H). tenuicornis* Parent, 1927 had no records of their distribution in Chinese provinces. *D.(H). rotundipennis* Loew, 1848 was newly recorded in Inner Mongolia and distributed in Hulun Buir and West Ujimqin Banner. The subgenus *Macrodolichopus* had two species distributed in China, namely *D.(M). diadema* Haliday, 1832 and *D.(M). obscuripes* Stackelberg, 1925, both of which are distributed in Qinghai province. The subgenus *Dolichopus* has many more species than the other two subgenera and they are distributed widely in China. When combined with the records from this study, the provinces in southern China had fewer records of the genus *Dolichopus* than those in northern China, where Inner Mongolia is located. With more studies, the distribution pattern of *Dolichopus* in China can be better clarified.

The *Dolichopus* species were widely distributed in Inner Mongolia. Every city had records of *Dolichopus* species except for Bayan Nur. At the same time, it seems that *Dolichopus* is adapted to three ecosystems in Inner Mongolia. For instance, it was found that *Dolichopus* were distributed in the mountains of the nature reserves in Inner Mongolia (Mount Jiufeng, Mount Helan, Saihanwula, Arxan, and Daqinggou) and the grasslands in Hulun Buir, Tongliao, Sonid Left Banner, Abaga, West Ujimqin Banner, and East Ujimqin Banner. Moreover, the wetlands in Hinggan League and Hulun Buir also recorded occurrences of *Dolichopus* species. However, the species level distribution patterns were quite different. Some species were distributed widely while some species only occurred in one place. For example, *D. longipilosus* Zhang et Yang, 2008, *D. bigeniculatus* Parent, 1926, *D. galeatus* Loew, 1871, *D. geniculatus* Stannius, 1831, *D. hilaris* Loew, 1862, *D. tewoensis* Yang, 1998 *D. zernyi* Parent, 1927 and *D. zhoui* Zhang, Yang et Grootaert, 2004 were only distributed in one location, whereas *D. agilis* Meigen, 1824, *D. linearis* Meigen, *D. martynovi* Stackelberg, 1930 1824, *D. plumitarsis* Fallén, 1823 and *D. longicornis* Stannius, 1831 were distributed in more than five locations. Considering the implication of these insects as natural enemies, further research on the distribution of *Dolichopus* species is needed.

The larvae of *Dolichopus* are aquatic and semi-aquatic while the adults appear in wet conditions, such as rivers, lakes, and forests [5,12]. The results of the MaxEnt model illustrated that the moderately, extremely, and highly suitable areas for *Dolichopus* were mainly distributed near water, such as in river basins (Figure 8). The largest extremely and highly suitable area was in Hulun Buir, which could be due to the vegetation or the river basins. The results also showed that the Helan and Yabulai Mountain Ranges (Alxa League) were extremely and highly suitable for the occurrence of *Dolichopus*. The Helan Mountains are steep on their east side, which obstructs the southeast monsoon when it moves westwards. The air current climbs upwards, resulting in orographic rain [50,51] and a large area with a semi-humid climate in the Helan Mountains. This could provide suitable climatic conditions for *Dolichopus*. Additionally, the Yabulai Mountains are located in temperate desert arid regions with no permanent rivers. However, due to the cover that is provided by the landform in the south valley, there is floating water in the Yabulai Mountains, which could be the reason for the occurrence of *Dolichopus*. Moreover, there were some extremely, highly, and moderately suitable areas in the Otingdag Sandy Land (Xilin Gol League), and there is a gradient of precipitation in the Otingdag Sandy Land which is relatively high in the east and relatively low in the west [52]. Furthermore, the Otingdag Sandy Land is a famous desert with many water holes and streams which could explain the occurrence of *Dolichopus*.

Generally, the potential geographic distribution (Figure 8) corresponded with the distribution pattern (Figure 1). However, with continuing investigations, insect distribution and diversity will become clearer. This study will provide basic data if *Dolichopus* species become the main predator with the changing of the weather.

## 5. Conclusions

This taxonomic study improved the knowledge of *Dolichopus* in Inner Mongolia. In this study, three new species and twelve newly recorded species were found in Inner Mongolia, which increased the number of *Dolichopus* species recorded in Inner Mongolia from eight to twenty-three. The number of the species of the genus in Inner Mongolia should increase as investigations continue. This study also indicated the potential distribution of *Dolichopus* in Inner Mongolia. Generally, the potential geographic distribution corresponded with the distribution records. Further research should focus on collecting more specimens near river basins, especially in Hulun Buir, as well as the sandy land in Xilin Gol League. Thus, the diversity of *Dolichopus* in Inner Mongolia was elucidated, and the potential distribution of the genus was provided. At the same time, the taxonomic study and potential geographic distribution provided basic data for further study.

## Data Availability

Data supporting results in this study can be obtained through contact with the first author (X.Y.).

## References

[1] Chen X., Dong S., Shi W., Ding W., Zhang Y., Li B., Shao Z., Wang Y. (2022). Construction of the Continental Asia in Phanerozoic: A Review. Acta Geol. Sin.—Engl. Ed..

[2] Cai X., Zhi D., Luo Z., Wu Y. (2011). Discovery and significance of pleistocene alluvial deposition in Guyang Basin northeast margin, Inner Mongolia. J. Stratigr..

[3] Sha Y., Shi Z., Liu X., An Z. (2015). Distinct impacts of the Mongolian and Tibetan Plateaus on the evolution of the East Asian monsoon. J. Geophys. Res. Atmos..

[4] Yang D., Zhu Y., Wang M., Zhang L. (2006). World Catalog of Dolichopodidae (Insecta: Diptera).

[5] Yang D., Zhang L., Wang M., Zhu Y. (2011). Fauna Sinica Insecta Vol. 53. Diptera Dolichopodidae.

[6] Negrobov O.P., Maslova O.O., Selivanova O.O. (2020). A new species of the genus *Dolichopus* Latr. (Dolichopodidae, Diptera) from Altal Republic and Mongolia. Acta Zool. Acad. Sci. Hung..

[7] Ulrich H. (2004). Predation by adult Dolichopodidae (Diptera): A review of literature with an annotated prey-predator list. Stud. Dipterol..

[8] Ulrich H., Schmelz R.M. (2001). Enchytraeidae as prey of Dolichopodidae, recent and in Baltic amber (Oligochaeta; Diptera). Bonn. Zool. Beitr..

[9] Yates M. (1984). The biology of the oak bark beetle, Scolytus intricatus (Ratzeburg)(Coleoptera: Scolytidae), in southern England. Bull. Entomol. Res..

[10] Kazerani F., Khaghanimia S., Grichanov I. (2013). Diversity of the genus Dolichopus Latreille in three different habitats of East Azerbaijan Province, with new records for Iran. Arx. Miscel Lània Zoològica.

[11] Iordosopol E. The role of the dolichopodidae and empididae families in the plum plantation and tritrophic relationships. Proceedings of the Simpozionul “Biotehnologii Avansate—Realizări şi Perspective”.

[12] Dyte C. (1959). Some interesting habitats of larval Dolichopodidae (Diptera). Entomol. Mon. Mag..

[13] Cumming J., Wood D.M., Kirk-Spriggs A.H., Sinclair B.J. (2017). Adult Morphology and Terminology. Manual of Afrotropical Diptera.

[14] Phillips S.J., Anderson R.P., Schapire R.E. (2006). Maximum entropy modeling of species geographic distributions. Ecol. Model..

[15] Graham C.H., Elith J., Hijmans R.J., Guisan A., Townsend Peterson A., Loiselle B.A. (2008). The influence of spatial errors in species occurrence data used in distribution models. J. Appl. Ecol..

[16] Zhang L., Yang D. (2008). New species of *Dolichopus* Latreille, 1796 from China (Diptera: Dolichopodidae). J. Nat. Hist..

[17] Meigen J.W. (1824). Systematische Beschreibung der Bekannten Europäischen Zweiflügeligen Insekten.

[18] Negrobov O.P., Rodionova S.Y., Maslova O.O., Selivanova O.V. (2005). Key to the males of the Palaearctic species of the genus Dolichopus Latr. (Diptera: Dolichopodidae). Int. J. Dipterol. Res..

[19] Negrobov O.P. (1973). Die Dolichopodiden-Arten (Diptera) aus der Mongolischen Volksrepublik. II. Acta Zool. Acad. Sci. Hung..

[20] Parent O. (1934). Espèces nouvelles de Diptères Dolichopodides. Encycl. Entomol. (B II) Diptera.

[21] Parent O. (1926). Dolichopodides nouveaux de l’extrême orient paléarctique. Encycl. Entomol. (B II) Diptera.

[22] Haliday A.H. (1832). The characters of two new dipterous genera, with indications of some generic subdivisions and several undescribed species of Dolichopodidae. Zool. J..

[23] Stackelberg A.A. (1930). Dolichopodidae. Die Fliegen der Palaearktischen Region.

[24] Loew H. (1871). Systematische Beschreibung der Bekannten Europäischen Zweiflugeligen Insecten. Beschreibung Europäische r Dipteren.

[25] Stannius F.H. (1831). Die europäische n Arten der Zweifluglergattung Dolichopus. Isis Von Oken.

[26] Parent O. (1928). Les Dolichopus paléarctiques, clé de détermination des mâles. Ann. Société Sci. Brux..

[27] Loew H. (1862). Sechs neue europaische Dipteren. Wien. Entomol. Monatschrift.

[28] Smirnov E.S. (1948). Materials for the fauna of Dolichopus of the Far East. Nauchno-Metod. Zap. Gl. Upr. Zapov..

[29] Zetterstedt J.W. (1843). Diptera Scandinaviae Disposita et Descripta.

[30] Zetterstedt J.W. (1849). Diptera Scandinaviae Disposita et Descripta.

[31] Stackelberg A.A. (1930). Résultats scientifiques de l’expédition hydrofaunistique du Musée Zoologique dans la Sibérie orientale en 1927. I. Diptera, Dolichopodidae. Part I. Genus Dolichopus Latr. Annuaire du Musée Zoologique de l’Academie des Sciences de l’URSS.

[32] Parent O. (1927). Contribution à l’étude de la distribution géographique de quelques espèces de Dolichopodides. Congrès des Sociétés Savantes.

[33] Scopoli J.A. (1763). Entomologia Carriolica Exhibens Insecta Carnioliae Indigene et Distributa in Ordines, Genera, Species, Varietates Methodo Linnaeana.

[34] Fallén C.F. (1823). Monographia Dolichopodum Sveciae. Lundae.

[35] Walker F. (1849). List of the Specimens of Dipterous Insects in the Collection of the British Museum.

[36] Van Duzee M.C. (1930). Three new Dolichopids from California and Colorado (Diptera). Pan-Pac. Entomol..

[37] Negrobov O.P. (1911). Family Dolichopodidae. Dolichopodidae-platypezidae.

[38] Yang D. (1998). New and little known species of Dolichopodidae from China (II). Bull. L’institut R. Sci. Nat. Belg..

[39] Parent O. (1927). Dolichopodides paléarctiques nouveaux ou peu connus. Encycl. Entomol. (B II) Diptera.

[40] Zhang L., Yang D., Grootaert P. (2004). Revision of the Dolichopus tewoensis group (Diptera, Dolichopodidae) from China. Biologia.

[41] Loew H. (1848). Dipterologisches. Stettin. Entomol. Ztg..

[42] Brooks S.E. (2005). Systematics and phylogeny of the Dolichopodinae (Diptera: Dolichopodidae). Zootaxa.

[43] Negrobov O.P., Grichanov I.Y., Barkalov A.V. (2009). The Dolichopus latipennis species group (= Hygroceleuthus Loew) in the Palearctic Region (Diptera: Dolichopodidae). Zootaxa.

[44] Chandler P. (1998). Checklists oflnsects ofthe British Isles (New Series). Part I: Diptera. Handbooks for the Identification of British Insects.

[45] Negrobov O., Stackelberg A. (1969). Faune des Dolichopodidae. Opredeliteli Nasekomych Evropeiskoi Ciasti SSSR.

[46] Van Duzee M.C., Curran C.H. (1934). Key to the males of Nearctic Dolichopus Latreille (Diptera). Am. Mus. Novit..

[47] Foote R.H. (1965). Family dolichopodidae. A Catalog of the Diptera of the America North of Mexico.

[48] Pollet M.A., Brooks S.E., Cumming J.M. (2004). Catalog of the Dolichopodidae (Diptera) of America north of Mexico. Bull. Am. Mus. Nat. Hist..

[49] Grichanov I.Y. (2016). A new species of Dolichopus from the Siberian Republic of Khakassia (Diptera: Dolichopodidae: Dolichopus latipennis species group). Amurian Zool. J..

[50] Ji X., Sang J., Yang K., Mu J., Chen X., Zhang N. (2009). Statistical forecast model for rainstorm circulation in eastern foot of Helan Mountain. J. Arid. Land Resour. Environ..

[51] Chen Y., Li J., Li X., Zhang S., Yang Y., Su Y., Yao S., Liu J. (2021). Spatio-temporal distribution of the rainstorm in the east side of the Helan Mountain and the possible causes of its variability. Atmos. Res..

[52] Qi D., Yang H., Lu Q., Gan H., Chu J. (2021). Types and characteristics of plant communities in the Otingdag Sandy Land. J. Desert Res..

